# Optimal Treatment for Intermediate- and High-Risk, Nonmuscle-Invasive Bladder Cancer

**DOI:** 10.1100/tsw.2006.403

**Published:** 2006-03-09

**Authors:** A.P.M. van der Meijden

**Affiliations:** Department of Urology, Jeroen Bosch Hospital, 5200 ME 's-Hertogenbosch, The Netherlands

**Keywords:** non-muscle invasive bladder cancer, prognostic factors

## Abstract

According to clinical and pathological factors the prognosis of a patient with non-muscle invasive bladder tumors can be assessed. The prognosis is determined by the likelihood of recurrence(30-70%) and/or progression to muscle invasive bladder cancer(1-15%).Trans urethral resection of bladder tumors remains the initial therapy but adjuvant intravesical instillations are necessary.All patients benefit from a single immediate post operative instillation with a chemotherapeutic agent and for low risk tumors this is the optimal therapy.Patients with intermediate and high risk tumors need more intravesical chemo-or immunotherapy. Chemotherapy reduces recurrences but not progression. Intravesical immunotherapy(BCG) prevents or delays progression. Patients at high risk for progression may need upfront cystectomy.

## INTRODUCTION

The term "superficial bladder cancer", used for many years in numerous publications, should be avoided, as it is inaccurate in its description to characterize so-called superficial bladder tumors. In the TNM system[1], Ta, T1, and carcinoma *in situ* (CIS) belong to the superficial bladder tumors, while T2, T3, and T4 are muscle-invasive neoplasms. However, while Ta and CIS are confined to the mucosa, being thus superficial, the biological behavior, prognosis, and treatment are quite different. T1 tumors do not infiltrate into the muscle wall of the bladder, but they penetrate into the lamina propria or submucosa, making them infiltrative tumors by definition.

## PROGNOSTIC FACTORS

Patients with nonmuscle-invasive bladder cancer may be divided into three risk groups: low, intermediate, and high[2]. Two events can occur in patients with nonmuscle-invasive tumors after treatment: they may recur or they may progress to muscle-invasive bladder tumors. While the likelihood of recurrence and progression is considerable in high-risk tumors, patients with intermediate-risk tumors have a far greater chance to encounter recurrence than progression[3,4].

The European Association of Urology guidelines committee on bladder cancer defined nonmuscle-invasive bladder tumors according to risk factors[5]. Low-risk tumors are single TaG1, <3 cm diameter. High-risk tumors are T1G3, multifocal, or highly recurrent and CIS. Intermediate risk: all other tumors Ta, T1, G1-2, multifocal, >3 cm diameter.

## TRANSURETHRAL RESECTION (TUR) OF TA, T1 BLADDER TUMORS

The gold standard for treating Ta, T1 tumors remains transurethral resection (TUR). CIS cannot be treated by TUR by its flat and multifocal nature. Small tumors can be resected in one chip where the chip contains the complete tumor plus a part of the underlying bladder wall. Larger tumors have to be resected in fractions. First, the exophytic tumor tissue is removed. Then, separately, the underlying bladder wall is resected into the muscle. Without the presence of muscle, the pathologist is unable to stage the tumor as Ta, T1, or T2. In case of large tumor(s), it is advised to also resect the edges of the resection area separately, as CIS may be present there. The chips of the three different resections have to be stored in three separate containers to enable the pathologists to make a correct diagnosis. Cauterization has to be avoided as much as possible to prevent tissue destruction. Necrotic and cauterized tissue also hampers a correct staging and grading. A complete and correct TUR is essential for the prognosis of the patient[5].

## BLADDER BIOPSIES

Bladder tumors are often multifocal. CIS, dysplasia, inflammation, etc. may present themselves as velvet-like, reddish areas in the bladder or may not be visible at all. If apart from a papillary tumor the rest of the bladder mucosa has a normal aspect and if urine cytology is negative, it is not advised to perform routine at-random biopsies. The likelihood of detecting CIS is extremely low and the choice of adjuvant intravesical therapy is not influenced by its result[6]. However, when the cytology is positive or when abnormal areas of urothelium are seen, it is advised to take cold-cup biopsies or biopsies with the resection loop. Guidelines on the diagnosis and treatment of CIS have been published separately.

## SECOND RESECTION

Although it seems an easy procedure to remove a bladder tumor by TUR, it has been demonstrated that the occurrence of a new tumor or the presence of residual tumor are frequently observed[7]. A second TUR should be performed when the initial resection was incomplete, e.g., when multiple and/or large tumors are present; furthermore, when a T1, high-grade tumor has been detected at the initial TUR. The likelihood that a T1G3 tumor is understaged, thus being a muscle-invasive tumor, is about 10%[7]. As the treatment of a T1G3 tumor and a T2 tumor is completely different, correct staging is important. It has been demonstrated that a second resection led to reduced recurrences and improved prognosis. There is no consensus on the timing of a second TUR, but most procedures are done between 2 and 6 weeks after the initial TUR.

## ONE IMMEDIATE POSTOPERATIVE INSTILLATION

Although a state-of-the-art TUR by itself could eradicate a Ta, T1 tumor completely, that type of tumor will recur in a high percentage and progress to muscle-invasive bladder cancer in a limited number of cases. Therefore, it is necessary to treat every patient adjuvantly with intravesical chemotherapy or immunotherapy (Bacille Calmette Guerin [BCG])[8]. A meta-analysis of 7 randomized trials including 1,476 patients with a median follow-up of 3.4 years demonstrated that one immediate instillation of chemotherapy after TUR decreases the relative risk of recurrence by 40%[8]. Both single and multiple tumors benefit from just one single instillation. The effect can be explained by destroying circulating tumor cells, immediately after TUR or as an ablative effect (chemoresection) of residual tumor cells at the resection site. The timing of the instillation is crucial. In all studies, the instillation was administered within 24 h. One study reported that if the first instillation was not given within 24 h, the risk of recurrence increased twofold. There is no superior drug with regard to efficacy. Mitomycin C, Epirubicin, and Andriamycin all have shown a beneficial effect. The same meta-analysis demonstrated that in every 100 patients, 12 TURs may be avoided using one postoperative instillation. This means that 8.5 patients must be treated to prevent one recurrence[8]. Since the costs of a TUR, anesthesia, and hospitalization exceed the costs of one instillation in most countries, this procedure is considered cost effective.

## EXTRA ADJUVANT INTRAVESICAL INSTILLATIONS

The need for further adjuvant intravesical therapy depends on the prognostic risk of the superficial bladder tumors. A single immediate instillation optimally reduces the recurrence rate in patients of the low-risk group[8] and may be considered as the standard treatment for these patients. For the other patients, however, it remains an incomplete treatment because the likelihood of recurrence and progression remains high. The effect of the immediate instillation of chemotherapy occurs during the first and second year[9]. It was calculated by the analysis of the data of 5 randomized trials that the reduction of recurrence lasts for a period of approximately 500 days. The choice between chemo- or immunotherapy largely depends on the risk that has to be reduced: recurrence or progression. In low-grade tumors, recurrence should be prevented and adjuvant chemotherapy bladder instillations are effective for this propose. When progression to a muscle-invasive tumor is expected in high-grade tumors, BCG therapy has proven to be clearly superior to intravesical chemotherapy. Two meta-analyses demonstrated that BCG prevents, or at least delays, progression[10,11]. A meta-analysis of European Organization for Research and Treatment of Cancer (EORTC) and Medical Research Council (MRC) data of their superficial bladder tumor trials, comparing intravesical chemotherapy vs. TUR alone, demonstrated that intravesical chemotherapy does not prevent progression[12].

## INTRAVESICAL CHEMOTHERAPY

No superiority of any chemotherapeutic agent over another has ever been demonstrated[13]. It remains controversial how long and how frequent the instillations have to be given. The EORTC demonstrated that administrating intravesical chemotherapy monthly for 1 year vs. monthly for a period of 6 months did not reduce the recurrence rate when the first instillation was given immediately after TUR[13]. This observation was confirmed by a Japanese study[14]. Another randomized trial reported reduced recurrence rates after 1 year of treatment with Epirubicin (19 instillations) compared to only 3 months (9 instillations)[15]. In view of these contradictory results, one cannot recommend long-term intravesical therapy. Chemical cystitis is the most frequently reported side effect with all drugs used. General side effects are very rare as reabsorption from the bladder is extremely low. Chemical cystitis is related to the intensity of the regimen (concentration and frequency). In most cases, it resolves after delaying or stopping treatment. An allergic skin reaction may occur on the hands and the genital region using Mitomycin C. This is a delayed type IV hypersensitivity reaction evoked in the bladder and not caused by spilling the drug on the skin.

## INTRAVESICAL BCG INSTILLATIONS

Although BCG is considered to be a very effective treatment, consensus exists that not every patient with superficial bladder cancer should be treated with BCG due to its greater risk of toxicity. Ultimately, the choice of treatment will depend on the patient's risk of recurrence and progression. A number of authors have proposed risk groups for these endpoints. Millan-Rodriguez et al. defined risk groups according to patients stage, grade, CIS, and number of tumors[3,4]. The optimal treatment for low-risk, TaG1 and solitary T1G1 lesions is complete TUR followed by one immediate instillation of a chemotherapeutic drug[16]. Based on median follow-up of 3.3 years, about one-third of these patients recur, but the risk for progression is approximately 0%. Thus BCG will not alter the natural course of the disease in low-risk patients and may be considered overtreatment once its side effects are taken into account. In patients with high-risk tumors for whom a cystectomy is not carried out, no controversy exists about how to treat these patients. In multiple T1G3 tumors, Ta T1G3 tumors with or without CIS, and CIS alone, where 15% or more of the patients will progress, the advantages of intravesical BCG are more pronounced than in intermediate risk patients who are at a lower risk of progression[10]. The treatment of the remaining intermediate-risk tumors (multifocal T1G1, TaG2, and single T1G2 tumors) is more controversial. It consists of complete TUR followed by intravesical chemotherapy or intravesical BCG. The major issue in intermediate-risk tumors is to prevent recurrence and progression, of which recurrence is by far the most frequent. Millan-Rodriguez et al. found that while about 45% of these patients will have a recurrence, the likelihood of progression to muscle-invasive disease in these patients is low, approximately 1.8%[3,4]. In choosing between chemotherapy and BCG, it should be kept in mind that intravesical BCG is superior to intravesical chemotherapy in reducing recurrences, but it is more toxic. BCG can delay or prevent progression to muscle-invasive disease. Although BCG is superior to chemotherapy in preventing recurrences, controversy existed until recently whether BCG could delay or prevent progression to muscle-invasive disease. Some studies appeared to show an effect, while most did not. Individual studies only have very limited power to show a difference since they observe too few events during a short follow-up period. The meta-analysis carried out by the EORTC has provided a clinically relevant answer to this question. A total of 24 randomized trials were identified with follow-up information on progression on 4,864 patients[10]; 3,967 (81.6%) had only papillary tumors and 896 (18.4%) had primary or concomitant CIS. Five different BCG strains were employed and in 20 out of the 24 trials, some form of BCG maintenance was used. In 4 trials, only a 6-week induction course was employed. Based on a median follow-up of 2.5 years and a maximum of 15 years, 260 out of 2,658 patients (9.8%) on BCG progressed compared to 304 out of 2,205 (13.8%) in the control groups (TUR alone, TUR plus intravesical chemotherapy, or TUR plus another immunotherapy), a reduction of 27% in the odds of progression on BCG (OR = 0.73, $p = 0.0001$). The size of the reduction is similar in patients with Ta, T1 papillary tumors and in those with CIS.

## UPFRONT CYSTECTOMY

There is no consensus on how to treat patients with the highest chance for progression. These patients have T1G3 papillary tumors often with concomitant CIS. While the conservative treatment exists of TUR, second TUR, and maintenance BCG instillations, the alternative treatment consists of upfront radical cystectomy. In such a way, the high chance of progression and metastases is prevented before it may harm the patient. On the other hand, about one-third of patients will lose their bladder unnecessarily, as they might have been cured by BCG[17]. Upfront cystectomy is often a matter of deciding between pros and cons. If the patient is young, otherwise healthy, but bearing a very high-risk tumor, upfront cystectomy will often be selected. Such a patient will undergo the operation with less likelihood of complications than an older, more fragile patient with considerable comorbidity. Furthermore, in the era of neo-bladder, the quality of life has improved after cystectomy. However, above all, it should be realized that BCG is no panacee for all high-risk tumors. Even after an initial complete response, progression may occur in the long term.

## CYSTECTOMY AFTER FAILURE OF INSTILLATION THERAPY

Failure of adjuvant intravesical therapy is ill defined. While progression to muscle-invasive disease is the trigger for cystectomy in most cases, there are other features that may indicate that intravesical instillations fail. For instance, the time to respond to intravesical therapy is not defined. While it is known that BCG immunotherapy needs some time to evoke an immune response, it is unknown how long the clinician may wait without jeopardizing the patient. Delaying cystectomy might lead to progression, metastases, and death from bladder cancer. Furthermore, the appearance of new superficial tumors every 3 months, the consequent TUR, the ongoing intravesical instillations, etc. may give rise to a bladder of such low quality in terms of capacity, urge, pain, etc. that, in many cases, a patient is better off having their bladder surgically removed[18].

## RECOMMENDATIONS FOR INTERMEDIATE-RISK TUMORS

1. Complete TUR.
2. Second TUR if complete resection is not achieved (optional).

3a. Adjuvant intravesical chemotherapy (drug optional), schedule optional although the schedule used should not exceed 1 year. The first instillations should be administered at once postoperatively.

3b. Adjuvant intravesical immunotherapy: drug BCG (full dose or reduced dose in case of side effects). Maintenance schedule: at least 1 year, optionally up to 3 years.

## RECOMMENDATIONS FOR HIGH-RISK TUMORS

The treatment for high risk Ta, T1, G3 with or without CIS, or for CIS (alone) consists of:

1. Complete TUR of papillary tumors (standard).
2. Second TUR after 4–6 weeks (recommended) followed by one immediate postoperative instillation with chemotherapy drug (recommended), drug optional.

3a. Adjuvant intravesical immunotherapy drug: BCG (full dose or reduced dose in case of side effects). Maintenance schedule: at least 1 year optionally to 3 years.

3b. Radical cystectomy plus urinary diversion up front (optional) or if no response to BCG therapy is achieved (standard).
